# Associations of Urinary Phytoestrogen Concentrations with Sleep Disorders and Sleep Duration among Adults

**DOI:** 10.3390/nu12072103

**Published:** 2020-07-16

**Authors:** Jing Sun, Hong Jiang, Weijing Wang, Xue Dong, Dongfeng Zhang

**Affiliations:** 1 Department of Epidemiology and Health Statistics, The School of Public Health of Qingdao University, 308 Ningxia Road, Qingdao 266071, China; sunjing1011@163.com (J.S.); wangwj793@126.com (W.W.); dongxue199411@126.com (X.D.); zhangdf1961@126.com (D.Z.); 2 Department of Physiology, Shandong Provincial Key Laboratory of Pathogenesis and Prevention of Neurological Disorders and State Key Disciplines: Physiology, School of Basic Medicine, Qingdao University, Qingdao 266071, China

**Keywords:** sleep disorders, sleep duration, urinary phytoestrogens, concentration–response, NHANES

## Abstract

Current evidence on the relationship of phytoestrogens with sleep is limited and contradictory. In particular, studies on individual phytoestrogens and sleep have not been reported. Thus, this study aimed to appraise the associations of individual phytoestrogens with sleep disorders and sleep duration. This cross-sectional study comprising 4830 adults utilized data from the National Health and Nutrition Examination Survey 2005–2010. Phytoestrogens were tested in urine specimens. Sleep disorders and sleep duration were based on a self-reported doctor’s diagnosis and usual sleep duration. The main analyses utilized logistic and multinomial logistic regression models and a restricted cubic spline. In the fully adjusted model, compared with tertile 1 (lowest), the odds ratios (95% confidence intervals (CIs)) of sleep disorders for the highest tertile of urinary concentrations of enterolactone, enterodiol, and O-desmethylangolensin were 0.64 (0.41–1.00), 1.54 (1.07–2.21), and 1.89 (1.26–2.85), respectively. Linear inverse, approximatively linear positive, and inverted L-shaped concentration–response relationships were found between enterolactone, enterodiol, and O-desmethylangolensin and sleep disorders, respectively. Compared with normal sleep (7–8 h/night), the relative risk ratio (RRR) (95% CI) of very short sleep for enterolactone was 0.56 (0.36–0.86), and the RRR (95% CI) of long sleep risk for genistein was 0.62 (0.39–0.99). Furthermore, negative associations of genistein with sleep disorders and enterolactone with long sleep risk, as well as positive associations of enterodiol with both long and very short sleep, were observed in the stratified analysis by age or gender. Finally, a notable finding was that urinary O-desmethylangolensin concentration was positively related to sleep disorders in both females aged 40–59 years and non-Hispanic Whites but inversely associated with sleep disorders in both females aged 60 years or over and other Hispanics. Our findings suggested that enterolactone and genistein might be beneficial for preventing sleep disorders or non-normal sleep duration among adults, and enterodiol might be adverse toward this goal. However, the association of O-desmethylangolensin with sleep disorders might be discrepant in different races and females of different ages.

## 1. Introduction

Sleep disorders, classified into insomnia, central disorders of hypersomnolence, sleep-related breathing disorders, parasomnias, sleep-related movement disorders, circadian rhythm sleep–wake disorders, and other sleep disorders [1], are common conditions and can seriously harm human health and quality of life [2]. Studies have found that poor sleep (short or long sleep duration, or other sleep problems) was associated with obesity, cardiovascular disease, diabetes, hypertension, cancer, and higher mortality [3,4,5,6,7,8]. Exploration of modifiable factors for reducing the risk of sleep disorders is urgently required. Estrogen has been a focus of attention due to its influences on the central nervous system that is involved in regulating sleep [9]. Randomized trials have revealed that estrogen therapy could reduce sleep disturbances and improve sleep quality [10,11,12]. Despite the significant beneficial effect on sleep, the use of estrogen therapy is still very cautious in virtue of its potentially serious health risks [13,14,15,16,17]. Therefore, as the naturally occurring mimetic agents of estrogen, phytoestrogens have aroused great interest in their possibility of being estrogen substitutes.

Phytoestrogens are a group of plant-derived bioactive compounds that have estrogenic and anti-estrogenic effects due to their structures resembling estradiol [18,19] and they are also considered to be endocrine disruptors [20]. The two principal groups of phytoestrogens comprise lignans and isoflavones. Lignans mainly originate from oilseeds, dried seaweeds, and whole-grain cereals, and are metabolized into enterolactone and enterodiol by bacteria in the colon [21,22]. Isoflavones, which are primarily derived from soya beans, soy products, and legumes, consist of genistin and daidzin, which can be hydrolyzed into genistein and daidzein, respectively, where daidzein is further metabolized into O-desmethylangolensin (O-DMA) or equol by gut microbiota [22]. Different individuals have different capabilities to produce phytoestrogens via microbial synthesis due to the complex interaction of the colonic environment with internal and external factors [23,24]; for example, only approximately 30–50% of individuals can produce equol via gut bacterial metabolism [25]. Additionally, these metabolites can also be directly obtained from some animal products, such as dairy [26,27].

Thus far, several trial studies have explored the relationship between total isoflavone supplementation and sleep in climacteric women or androgen-deprived prostate cancer patients, with mixed findings, including improvement of sleep problems [28,29,30,31], no significant association [32,33,34], and even insomnia aggravation [35]. Only two observational studies have evaluated the association of total isoflavone consumption with sleep status among general adults and results showed that total isoflavone consumption was positively related to sleep quality and optimal sleep duration in Japanese adults and inversely associated with long sleep duration in Chinese adults [36,37]. However, no epidemiological study to date has appraised the relationship between lignans and sleep. Only several animal experiments have found the sedative and hypnotic effects of the lignan component [38,39]. Moreover, individual phytoestrogens have unequal biological activities and estrogen receptor (ER) affinities [40,41]. Studies have reported divergent associations between individual phytoestrogens and disease [42,43,44,45,46]. Thus, individual phytoestrogens may also have diverse impacts on sleep, and investigating the relationships with sleep among individual phytoestrogens may be more meaningful. Meanwhile, investigating phytoestrogens via dietary evaluation made it difficult to include all food origins and did not take into account the metabolic transformation of intestinal flora, causing inexact individual exposure; therefore, it is necessary to assess phytoestrogens based on biomarkers to reflect the true exposure. Additionally, the concentration–response relationships of phytoestrogens with sleep were also unknown. Therefore, the present study aimed to: first, appraise the associations between the urinary concentrations of individual phytoestrogens and sleep disorder risk among U.S. adults by utilizing data from the National Health and Nutrition Examination Survey (NHANES) 2005–2010; second, to explore the concentration–response relationships of them; third, to explore the gender, age, and race differences in the associations; and finally, to evaluate the associations of individual phytoestrogens with sleep duration.

## 2. Materials and Methods

### 2.1. Study Population

The NHANES is a cross-sectional, complex, multistage, and stratified probability sampling design representing the non-institutionalized U.S. civilian population, which aims to investigate the health status and nutrition condition of Americans [47,48]. The NHANES collects data via examinations implemented in the mobile examination center (MEC) and via household interviews. All of the participants provided informed consent and the protocol of investigation was authorized by the Research Ethics Review Board of the National Center for Health Statistics.

**Figure 1:**
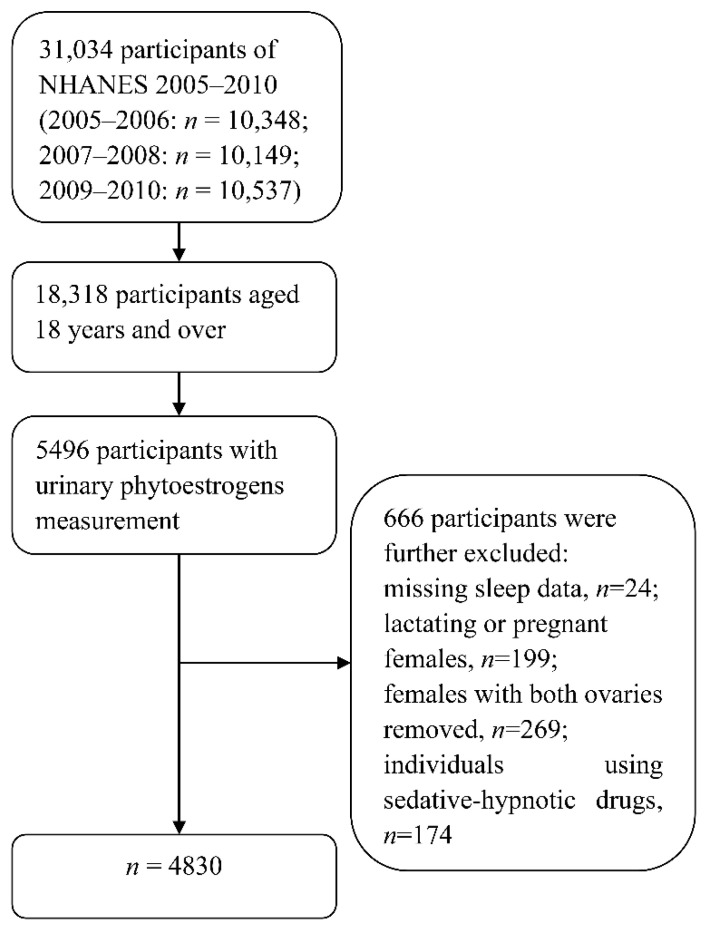
Flowchart of the screening process of eligible participants from the National Health and Nutrition Examination Survey 2005–2010.

This study chose three-cycle data (NHANES 2005–2006, 2007–2008, and 2009–2010) to create the current sample because the data of sleep and urinary phytoestrogens were measured simultaneously only in the three cycles. A total of 31,034 individuals were enrolled in the NHANES 2005–2010, where the number of participants aged 18 years and over was 18,318. A subsample of approximately one-third of all NHANES participants aged six years or over was chosen to measure urinary phytoestrogens, leaving 5496 participants. Among them, 666 individuals were further ruled out, including participants with missing sleep data (*n* = 24), lactating or pregnant females (*n* = 199), females with both ovaries removed (*n* = 269), and individuals using sedative-hypnotic drugs (*n* = 174). Ultimately, 4830 participants (age ≥ 18 years) with phytoestrogen data were analyzed in the current study (Figure 1).

### 2.2. Urinary Phytoestrogens Measurement

Spot urine specimens were collected in the MEC and urinary concentrations of individual phytoestrogens (enterolactone, enterodiol, daidzein, O-DMA, equol, and genistein) were tested in the one-third subsample of all NHANES participants aged six years or over by utilizing high-performance liquid chromatography–atmospheric pressure photoionization–tandem mass spectrometry during the NHANES 2005–2010. More details are available in the laboratory procedure manual [49]. Studies have revealed that phytoestrogen concentrations in spot urine are reliable biomarkers for phytoestrogen intake [23,50,51].

To correct for urine dilution, phytoestrogen concentrations were creatinine-standardized and expressed as μg/g creatinine [52]. Urine creatinine was measured by utilizing the Beckman CX3 during 2005–2006 but the Roche ModP was used since 2007; therefore, we adjusted creatinine 2005–2006 for the comparability of creatinine between 2005–2006 and 2007–2010 using the equations recommended by the NHANES [53].

### 2.3. Sleep Disorders and Sleep Duration Assessments

The sleep disorder investigations were administered by utilizing a computer-assisted personal interviewing system by trained interviewers in the home. Participants were classified into the sleep disorder groups based on a self-reported doctor diagnosis, and this classification method was used by prior published studies [54,55]. Sleep duration was categorized as 7–8 h/night (normal sleep), <5 h/night (very short sleep), 5–6 h/night (short sleep), and ≥9 h/night (long sleep) based on the self-reported usual sleep duration at night [54,56].

### 2.4. Covariates

The covariates gender, age, marital status, race, occupation, family income, educational level, body mass index (BMI), smoking status, alcohol consumption, use of female hormones, physical activity, caffeine intake, C-reactive protein, hypertension, depressive symptoms, and diabetes were chosen based on previous literature to control for potential confounding effects [12,36,57]. The details of the classification and criteria for covariates are displayed in Table S1.

### 2.5. Statistical Analysis

To explicate the complexity of the sampling design and create the estimated values of national representativeness, a primary sampling unit, strata information, and specific sampling weights for the one-third subsample were utilized in the present analyses. According to the analytical guideline [58], the new six-year weights were generated by dividing the two-year environmental weights by three and were applied in this study due to the combination of three two-year NHANES cycles.

The distribution types of continuous variables were identified using Kolmogorov–Smirnov normality tests. Numbers (percentages) and medians (interquartile ranges) were used to describe the qualitative data and non-normal quantitative data, respectively. Chi-square tests and Mann–Whitney *U* tests were performed to compare percentages for qualitative data and averages for non-normal quantitative data between the non-sleep disorders group and the sleep disorders group, respectively. Urinary concentrations of individual phytoestrogens were segmented into tertiles based on their distributions in the present population, with tertile 1 (lowest) being the referent. First, logistic regression analyses were conducted to appraise the relationships between urinary phytoestrogens and sleep disorders, along with calculating the odds ratios (ORs) and 95% confidence intervals (CIs). Six phytoestrogens were entered into model 1 simultaneously for controlling for potentially confounding effects. Model 2 additionally adjusted for gender and age, and model 3 was further adjusted for marital status, race, occupation, family income, educational level, BMI, smoking status, alcohol consumption, use of female hormones, physical activity, caffeine intake, C-reactive protein, hypertension, depressive symptoms, and diabetes. The concentration–response relationships of urinary phytoestrogens with sleep disorders were evaluated using restricted cubic spline functions of three knots (the 25th, 50th, and 75th percentiles of the exposure distributions) in model 3. Second, multinomial logistic regressions were utilized to evaluate the associations between urinary phytoestrogens and sleep duration in model 3, with normal sleep (7–8 h/night) being the referent. Third, considering the significant differences in sleep between different age groups and genders [59,60], we conducted stratified analyses by age (18–39, 40–59, and ≥60 years) and gender, as well as by age group separately for females and males, respectively. Finally, given that the intestinal metabolism of phytoestrogens may differ depending on the race [61], a stratified analysis by race (Mexican American, non-Hispanic Whites, non-Hispanic Blacks, and other Hispanics) was also performed. Statistical analyses were performed utilizing Stata 15.0 (Stata Corporation, College Station, TX, USA), and *p* < 0.05 (two-sided) suggested statistical significance.

## 3. Results

The characteristics of the participants in this study across sleep disorders are displayed in Table 1. In total, 4830 eligible individuals were analyzed, and the prevalence of sleep disorders was 6.25%. Except for family income and alcohol consumption, other characteristics were significantly different between the sleep disorders group and the non-sleep-disorders group. Individuals with the following characteristics were more likely to experience sleep disorders: older, male, non-Hispanic White, married or living with a partner, smoker, obese, hypertension, depressive symptoms, diabetes, higher education level, less physical activity, higher C-reactive protein concentration, higher caffeine intake, without work, and women using female hormones. Except for family income, alcohol consumption, and smoking status, other characteristics were significantly different between the male sleep disorders group and the male non-sleep-disorders group. However, there were significant differences between the female sleep disorders group and the female non-sleep-disorders group only in terms of age, BMI, use of female hormones, hypertension, depressive symptoms, and diabetes.

Table 2 presents the weighted ORs with 95% CIs for sleep disorders according to the tertiles of urinary phytoestrogen concentrations. In model 1, the urinary concentrations of enterolactone and genistein were inversely associated with sleep disorders, while the urinary O-DMA concentration was positively related to sleep disorders. After an additional adjustment for gender and age in model 2, the results were concordant with model 1. In the fully adjusted model (model 3), the negative association between the genistein concentration and sleep disorders was no longer significant, and the enterolactone concentration was still inversely associated with sleep disorders, while the urinary concentrations of enterodiol and O-DMA were positively related to sleep disorders. Compared with tertile 1 (lowest), the fully adjusted ORs (95% CIs) for sleep disorders for the highest tertile of urinary concentrations of enterolactone, enterodiol, and O-DMA were 0.64 (0.41–1.00), 1.54 (1.07–2.21), and 1.89 (1.26–2.85), respectively.

**Figure 2:**
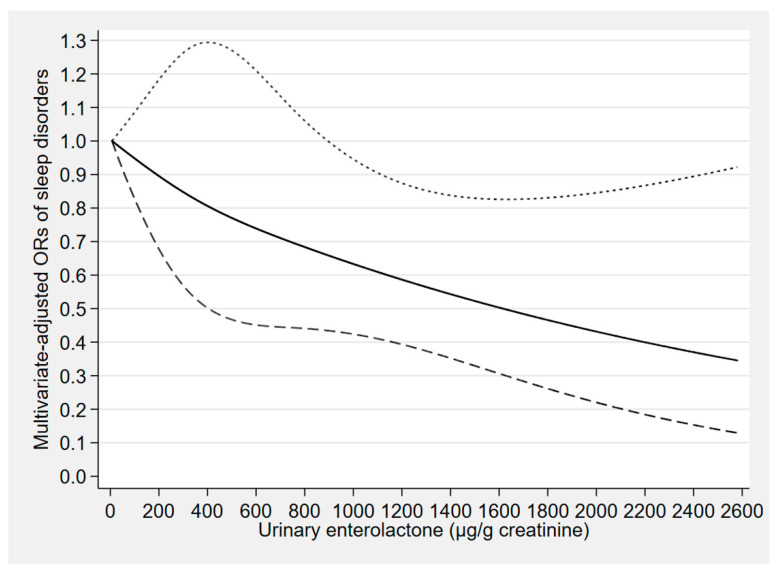
Concentration–response relationship of the urinary enterolactone concentration with sleep disorders. The solid line represents the estimated odds ratios (ORs) and the dashed lines represent their 95% confidence intervals. The relationship was adjusted for gender, age, marital status, race, occupation, family income, educational level, body mass index, smoking status, alcohol consumption, use of female hormones, physical activity, caffeine intake, C-reactive protein, hypertension, depressive symptoms, diabetes, and the other five phytoestrogens (tertiles).

**Figure 3:**
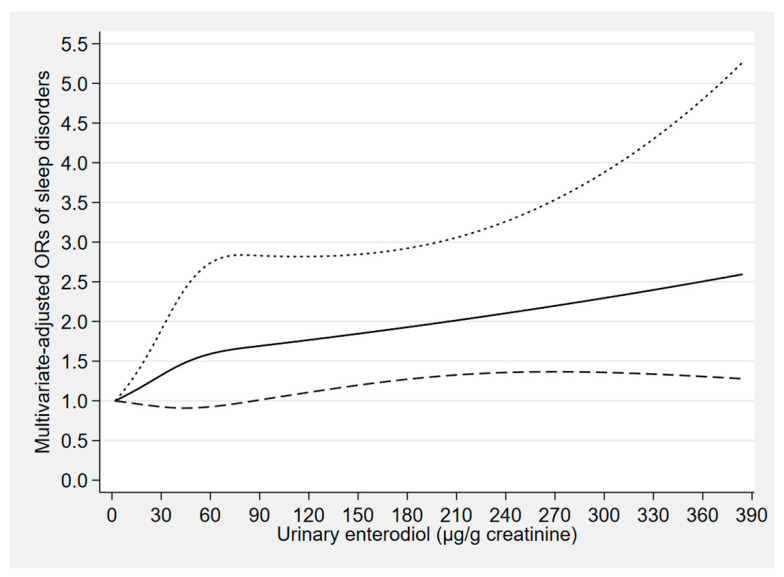
Concentration–response relationship of the urinary enterodiol concentration with sleep disorders. The solid line represents the estimated odds ratios (ORs) and the dashed lines represent their 95% confidence intervals. The relationship was adjusted for gender, age, marital status, race, occupation, family income, educational level, body mass index, smoking status, alcohol consumption, use of female hormones, physical activity, caffeine intake, C-reactive protein, hypertension, depressive symptoms, diabetes, and the other five phytoestrogens (tertiles).

**Figure 4:**
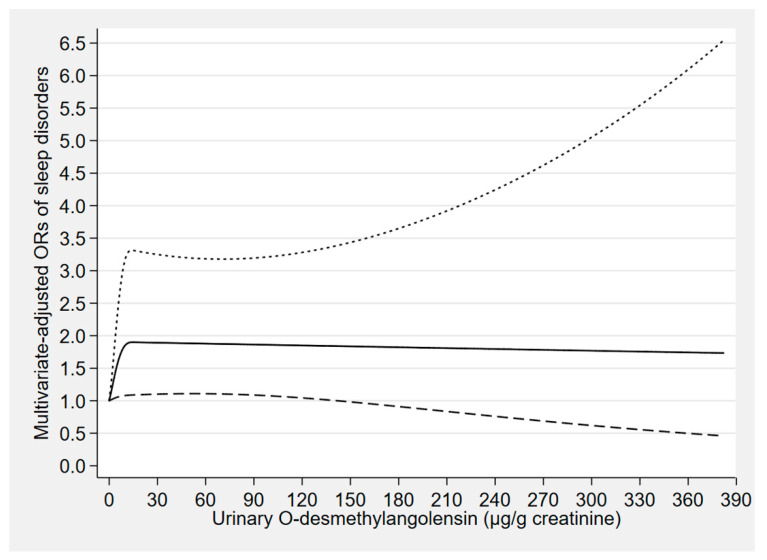
Concentration–response relationship of the urinary O-desmethylangolensin concentration with sleep disorders. The solid line represents the estimated odds ratios (ORs) and the dashed lines represent their 95% confidence intervals. The relationship was adjusted for gender, age, marital status, race, occupation, family income, educational level, body mass index, smoking status, alcohol consumption, use of female hormones, physical activity, caffeine intake, C-reactive protein, hypertension, depressive symptoms, diabetes, and the other five phytoestrogens (tertiles).

The concentration–response relationships of the urinary concentrations of enterolactone, enterodiol, and O-DMA with sleep disorders are depicted in Figure 2, Figure 3 and Figure 4, respectively. The urinary enterolactone concentration was linearly negatively associated with sleep disorders (*p*-nonlinearity = 0.849). The association began to show statistical significance when the enterolactone concentration reached around 904 μg/g creatinine (OR: 0.66, 95% CI: 0.43–0.99) (Figure 2). However, an approximately linear positive relationship was observed between the urinary enterodiol concentration and sleep disorders (*p*-nonlinearity = 0.274), and when the enterodiol concentration reached around 86 μg/g creatinine (OR: 1.68, 95% CI: 1.00–2.83), the relationship began to present statistical significance (Figure 3). There was a nonlinear positive (inverted L-shaped) association between urinary O-DMA concentration and sleep disorders (*p*-nonlinearity = 0.033). The OR of sleep disorders increased with increasing urinary O-DMA concentrations, and it arrived at a plateau when the O-DMA concentration was above approximately 13 μg/g creatinine (OR: 1.90, 95% CI: 1.09–3.31) (Figure 4).

**Figure 5:**
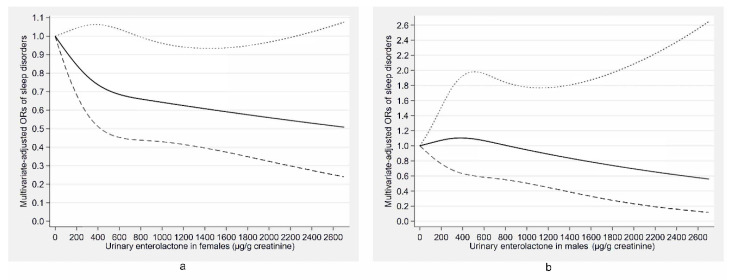
Concentration–response relationship of the urinary enterolactone concentration with sleep disorders for females (**a**) and males (**b**). The solid line represents the estimated odds ratios (ORs) and the dashed lines represent their 95% confidence intervals. The relationship adjusted for age, marital status, race, occupation, family income, educational level, body mass index, smoking status, alcohol consumption, use of female hormones (only in females), physical activity, caffeine intake, C-reactive protein, hypertension, depressive symptoms, diabetes, and the other five phytoestrogens (tertiles).

**Figure 6:**
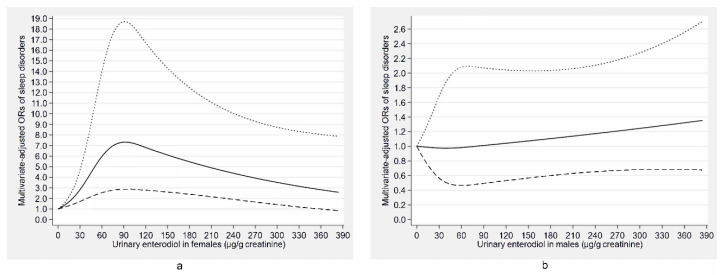
Concentration–response relationship of the urinary enterodiol concentration with sleep disorders for females (**a**) and males (**b**). The solid line represents the estimated odds ratios (ORs) and the dashed lines represent their 95% confidence intervals. The relationship adjusted for age, marital status, race, occupation, family income, educational level, body mass index, smoking status, alcohol consumption, use of female hormones (only in females), physical activity, caffeine intake, C-reactive protein, hypertension, depressive symptoms, diabetes, and the other five phytoestrogens (tertiles).

The associations of urinary phytoestrogen concentrations with sleep disorders stratified by age and gender are shown in Table 3 and Table 4, respectively. In the fully adjusted model, the urinary enterolactone concentration was still inversely associated with sleep disorders in females (OR: 0.45, 95% CI: 0.21–0.94). Meanwhile, the urinary enterodiol concentration was still positively related to sleep disorders among middle-aged adults (40–59 years) (OR: 2.56, 95% CI: 1.12–5.84) and females (OR: 3.79, 95% CI: 1.83–7.87), and the O-DMA concentration was still positively associated with sleep disorders in young adults (18–39 years) (OR: 4.16, 95% CI: 1.58–10.97). Additionally, there was a negative association between the genistein concentration and sleep disorders in middle-aged adults (40–59 years) (OR: 0.34, 95% CI: 0.13–0.88). There were no significant associations between the urinary phytoestrogen concentrations and sleep disorders in males and older adults (≥60 years). To further describe the gender difference in the associations of the urinary concentrations of enterolactone and enterodiol with sleep disorders, the concentration–response relationships of them for males and females are presented in Figure 5a,b and Figure 6a,b, respectively. The urinary enterolactone concentration was linearly negatively associated with sleep disorders in females (*p*-nonlinearity = 0.283) (Figure 5a) and the enterodiol concentration was positively related to sleep disorders for females in a nonlinear manner (*p*-nonlinearity < 0.001) (Figure 6a), whereas the associations were not significant in males (Figure 5b and Figure 6b).

Furthermore, the associations between the urinary phytoestrogen concentrations and sleep disorders stratified by age group separately for males and females are shown in Table 5. The urinary O-DMA concentration was positively associated with sleep disorders in males aged 18–39 years (OR: 6.57, 95% CI: 2.06–20.99) and females aged 40–59 years (OR: 15.14, 95% CI: 2.99–76.65), whereas the O-DMA concentration was inversely related to sleep disorders in females aged 60 years or over (OR: 0.25, 95% CI: 0.10–0.58). The urinary enterodiol concentration was positively related to sleep disorders in females aged 18–39 (OR: 6.52, 95% CI: 1.69–25.23) and 40–59 years (OR: 13.66, 95% CI: 2.06–90.70). Furthermore, the urinary equol concentration was inversely associated with sleep disorders in females aged 18–39 years (OR: 0.08, 95% CI: 0.01–0.84), and daidzein concentration was positively related to sleep disorders in females aged 60 years or over (OR: 10.67, 95% CI: 1.88–60.44).

The associations between the urinary phytoestrogen concentrations and sleep disorders stratified by race are displayed in Table 6. An interesting finding was that the urinary O-DMA concentration was positively related to sleep disorders in non-Hispanic Whites (OR: 2.16, 95% CI: 1.31–3.55) but inversely associated with sleep disorders in other Hispanics (OR: 0.13, 95% CI: 0.02–0.86). Furthermore, the urinary enterolactone concentration was negatively related to sleep disorders (OR: 0.56, 95% CI: 0.33–0.95), while the enterodiol concentration was positively associated with sleep disorders (OR: 1.89, 95% CI: 1.13–3.13) in non-Hispanic Whites and the equol concentration was positively related to sleep disorders in other Hispanics (OR: 3.22, 95% CI: 1.11–9.30).

Table 7 presents the weighted relative risk ratios (RRRs) with 95% CIs of sleep duration according to tertiles of the urinary phytoestrogen concentrations. In the fully adjusted model, compared with normal sleep (7–8 h/night), the urinary enterolactone concentration was negatively associated with very short sleep (<5 h/night) (RRR: 0.56, 95% CI: 0.36–0.86), and the genistein concentration was inversely related to a long sleep risk (≥9 h/night) (RRR: 0.62, 95% CI: 0.39–0.99).

The associations of urinary phytoestrogen concentrations with sleep duration stratified by age and gender are shown in Table 8 and Table 9, respectively. In the fully adjusted model, compared with normal sleep, the urinary enterolactone concentration was still negatively associated with very short sleep among young adults (18–39 years) (RRR: 0.24, 95% CI: 0.09–0.61) and a long sleep risk among older adults (≥60 years) (RRR: 0.49, 95% CI: 0.29–0.84). The urinary genistein concentration was still inversely associated with a long sleep risk in middle-aged adults (40–59 years) (RRR: 0.10, 95% CI: 0.02–0.56) and males (RRR: 0.51, 95% CI: 0.30–0.87). The urinary enterodiol concentration was positively related to very short sleep among young adults (18–39 years) (RRR: 2.73, 95% CI: 1.20–6.21) and a long sleep risk in females (RRR: 2.14, 95% CI: 1.11–4.13).

## 4. Discussion

To our knowledge, the current study was the first evaluation of the associations between the urinary concentrations of individual phytoestrogens (enterolactone, enterodiol, daidzein, O-DMA, equol, and genistein) and sleep disorders and sleep duration among general American adults. This study was based on data from the NHANES 2005–2010 and found that the urinary enterolactone concentration was linearly inversely associated with the risk of sleep disorders, while the enterodiol concentration was positively related to sleep disorders in an approximately linear manner and the O-DMA concentration was positively associated with sleep disorders in a nonlinear (inverted L-shaped) manner. The urinary enterolactone concentration was negatively associated with very short sleep, and the genistein concentration was inversely related to a long sleep risk. Furthermore, a negative association of the urinary genistein concentration with sleep disorders was observed in middle-aged adults (40–59 years). The urinary enterolactone concentration was inversely associated with a long sleep risk among older adults (≥60 years), while the enterodiol concentration was positively related to a long sleep risk in females and very short sleep in young adults (18–39 years). Finally, an interesting finding was that the urinary O-DMA concentration was positively related to sleep disorders in both females aged 40–59 years and non-Hispanic Whites, but was inversely associated with sleep disorders in both females aged 60 years or over and other Hispanics.

There has been no epidemiological study that has appraised the relationship of total lignans or individual lignans (enterolactone or enterodiol) with sleep disorders to date. However, animal experiments have indicated that a lignan component could increase sleep duration and decrease sleep latency via modulating the γ-aminobutyric acid (GABA)-ergic system [38,39], which supports our findings that enterolactone was negatively associated with sleep disorders and very short sleep, and enterodiol was positively related to a long sleep risk in females. We also found that enterolactone was inversely associated with a long sleep risk among older adults (≥60 years), while enterodiol was positively associated with sleep disorders in the whole population and very short sleep in young adults (18–39 years). This seems to suggest that enterolactone might be beneficial for preventing both long and very short sleep, whereas enterodiol might be adverse toward this goal. However, further comparisons were difficult due to limited prior studies.

Some studies have evaluated the association between total isoflavone consumption and sleep but the results were contradictory. A study with a cross-sectional design performed in Japanese adults found that the intake of isoflavone was positively related to sleep quality and optimal sleep duration [36]. Another longitudinal study of Chinese adults reported that isoflavone intake was inversely related to falling asleep in the daytime in females and long sleep duration in both genders [37], which was similar to our result regarding genistein and a long sleep risk. Several trial studies also showed that isoflavone supplementation alleviated insomnia or sleep disorders among climacteric women [28,29,30,31]. However, other trial studies of isoflavone supplementation in climacteric women or androgen-deprived males found no significant improvement in insomnia or sleep quality [32,33,34]. Meanwhile, another randomized, placebo-controlled, double-blinded trial over six months performed on climacteric women indicated that insomnia was more frequent in the isoflavone supplementation group [35], and the longitudinal study reported that soy milk (one of the main food sources for isoflavone intake in the study) was positively related to falling asleep in the daytime among males [37]. Similarly, we found a positive association between O-DMA and sleep disorders.

Thus far, little is known about individual isoflavones (daidzein, O-DMA, equol, and genistein) and sleep. The current study found discrepant associations of them with sleep disorders, where O-DMA was positively associated with sleep disorders, and genistein was negatively associated with sleep disorders in middle-aged adults (40–59 years). Likewise, there were also differential relationships between them and sleep duration, where only genistein was significantly inversely related to a long sleep risk, and no significant associations were found between daidzein, O-DMA, and equol and sleep duration in this study. Variable abilities in different individuals regarding the metabolic transformation of isoflavones [23], diverse biologic activities, and ER affinities of these metabolites (O-DMA and equol), as well as other individual isoflavones (daidzein and genistein) [40,41], and discrepant associations between them and sleep disorders or sleep duration may partially lead to the contradictory findings of prior studies focusing only on total isoflavones. Furthermore, potential gender, age, and race differences regarding these associations may also partially contribute to the prior contradictory findings.

The underlying mechanisms of the associations of phytoestrogens with sleep are unclear, where the following are several possible explanations. Estrogen can influence the synthesis and transport of serotonin [62] that is involved in the regulation of wakefulness and sleep [63], and studies have revealed that estrogen therapy alleviates sleep disturbances and improves sleep quality [10,11]. Therefore, phytoestrogens may affect the sleep–wake cycle through their estrogenic or anti-estrogenic effects [19], which may also explain the discrepant relationships of individual phytoestrogens with sleep disorders or sleep duration. A notable finding in our study was that the urinary O-DMA concentration was positively related to sleep disorders in females aged 40–59 years but was inversely associated with sleep disorders in females aged 60 years or over, which may be partly attributed to the anti-estrogenic and estrogenic effects of phytoestrogens, depending on the level of endogenous estrogen [64]. We also found that the urinary O-DMA concentration was positively related to sleep disorders in non-Hispanic Whites but was inversely associated with sleep disorders in other Hispanics, which may be partially due to the race difference in the compositions of gut microbiota and daidzein-metabolizing phenotypes [61,65]. Equol has estrogenic activities [66], which may partly contribute to the inverse association of equol with sleep disorders in females aged 18–39 years, while the mechanisms for the positive association between equol and sleep disorders in other Hispanics need to be investigated. Furthermore, the inter-individual variation of phytoestrogen metabolism by gut microbiota [67] may also contribute to the complexity of these results. The above-mentioned mechanisms may partly explain our findings, and further studies on the related mechanisms remain necessary.

This study has several advantages. First, the phytoestrogen assessments were based on urinary biomarkers, which reflected all food origins of phytoestrogens and took into account the metabolic transformation of intestinal flora, representing true and biologically effective exposures. Second, the relationships with sleep disorders or sleep duration were evaluated for individual phytoestrogens (enterolactone, enterodiol, daidzein, O-DMA, equol, and genistein), which made up for the fact that previous epidemiological studies did not investigate the relationship of lignans with sleep and focused only on total isoflavones while ignoring the potential differences of individual isoflavones. Third, our findings were based on data from the NHANES, which was carefully designed, of high quality, and nationally representative. Fourth, concentration–response relationships between individual phytoestrogens and sleep disorders were appraised in this study. Fifth, we further explored gender, age, and race differences in the associations of individual phytoestrogens with sleep disorders. Additionally, we adjusted for a wide range of confounders to control for potential confounding bias.

However, several potential limitations should also be considered. First of all, the cross-sectional design precludes the possibility of causal inference. Second, although phytoestrogen concentrations in spot urine are reliable biomarkers for phytoestrogen intake [23,50,51], it might be difficult to accurately reflect long-term intake information because spot urine was collected only once. Nonetheless, studies have demonstrated that phytoestrogen concentrations in spot urine are relatively stable and significantly related to dietary phytoestrogen intake over the long term [68,69]. Third, the sleep disorders assessment was via a self-reported doctor diagnosis, which might involve recall bias, and self-reported usual sleep duration might be not objective enough; however, objective sleep measurement, such as polysomnography, may be difficult to implement in large-scale surveys. Fourth, although three-cycle data with national representativeness were combined and the sample was relatively large, the case group might still be not enough due to the lower prevalence, which might lead to bias. Furthermore, although we adjusted multiple covariates, measurement errors and other factors affecting sleep quality might influence the present findings. However, the present results were approximate in the three models, suggesting that the results might be robust and exposure factors might be independently associated with sleep. Finally, we could not further explore the relationships between phytoestrogens and specific types of sleep disorders in virtue of the limited sleep disorders data.

## 5. Conclusions

The current findings suggested that the urinary enterolactone concentration was linearly inversely associated with the risk of sleep disorders among American adults, whereas the enterodiol and O-DMA concentrations were positively related to sleep disorders in approximately linear and inverted L-shaped manners, respectively. Meanwhile, the urinary enterolactone concentration was negatively associated with very short sleep and the genistein concentration was inversely related to a long sleep risk. Furthermore, a negative association of urinary genistein concentration with sleep disorders was observed in middle-aged adults. The urinary enterolactone concentration was inversely associated with a long sleep risk among older adults, while the enterodiol concentration was positively related to a long sleep risk in females and very short sleep in young adults. Finally, a notable finding was that the association of the urinary O-DMA concentration with sleep disorders was different between females aged 40–59 years and females aged 60 years or over, as well as between non-Hispanic Whites and other Hispanics. It may be meaningful and vital to choose more specific individual phytoestrogens, not choosing total lignans or isoflavones for preventing or improving sleep problems, and to match target groups given the potentially differential associations of individual phytoestrogens with sleep, as well as the gender, age, and race differences in the associations. Prospective cohort or trial studies evaluating the relationships of individual phytoestrogens with sleep are warranted to confirm the current findings.

## Figures and Tables

**Table 1:** Characteristics of the subjects (age ≥ 18 years) by sleep disorders (National Health and Nutrition Examination Survey 2005–2010).

Characteristics	Total			Males		Females			
	No Sleep Disorders	Sleep Disorders	*p*-Value	No Sleep Disorders	Sleep Disorders	*p*-Value	No Sleep Disorders	Sleep Disorders	*p*-Value
Number of individuals (%)	4528 (93.75)	302 (6.25)		2396 (92.80)	186 (7.20)		2132 (94.84)	116 (5.16)	
Gender (%)			0.003			-			-
Female	2132 (47.08)	116 (38.41)		-	-		-	-	
Male	2396 (52.92)	186 (61.59)		-	-		-	-	
Age (%)			<0.001			<0.001			0.005
18–39 years	1846 (40.77)	71 (23.51)		957 (39.94)	40 (21.51)		889 (41.70)	31 (26.72)	
40–59 years	1378 (30.43)	109 (36.09)		707 (29.51)	66 (35.48)		671 (31.47)	43 (37.07)	
≥60 years	1304 (28.80)	122 (40.40)		732 (30.55)	80 (43.01)		572 (26.83)	42 (36.21)	
Race (%)			<0.001			<0.001			0.287
Mexican American	914 (20.19)	39 (12.91)		466 (19.45)	16 (8.60)		448 (21.01)	23 (19.83)	
Non-Hispanic White	2040 (45.05)	173 (57.28)		1118 (46.66)	116 (62.37)		922 (43.25)	57 (49.14)	
Non-Hispanic Black	975 (21.53)	54 (17.88)		529 (22.08)	34 (18.28)		446 (20.92)	20 (17.24)	
Other Hispanic	385 (8.50)	28 (9.27)		180 (7.51)	14 (7.53)		205 (9.62)	14 (12.07)	
Other race	214 (4.73)	8 (2.65)		103 (4.30)	6 (3.23)		111 (5.21)	2 (1.72)	
Education (%)			0.006			0.004			0.444
High school	1117 (24.69)	63 (20.86)		619 (25.86)	41 (22.04)		498 (23.37)	22 (18.97)	
Below high school	1317 (29.10)	71 (23.51)		708 (29.57)	39 (20.97)		609 (28.58)	32 (27.59)	
Above high school	2091 (46.21)	168 (55.63)		1067 (44.57)	106 (56.99)		1024 (48.05)	62 (53.45)	
Occupation (%)			0.002			<0.001			0.626
No work	1878 (41.48)	156 (51.66)		898 (37.49)	100 (53.76)		980 (45.97)	56 (48.28)	
Regular night or evening shift/rotating shift/other	735 (16.24)	41 (13.58)		428 (17.87)	22 (11.83)		307 (14.40)	19 (16.38)	
Regular daytime schedule	1914 (42.28)	105 (34.77)		1069 (44.63)	64 (34.41)		845 (39.63)	41 (35.34)	
Family income/year (%)			0.721			0.932			0.536
$20,000 and over	3019 (74.07)	201 (73.09)		1630 (75.74)	126 (75.45)		1389 (72.19)	75 (69.44)	
Below $20,000	1057 (25.93)	74 (26.91)		522 (24.26)	41 (24.55)		535 (27.81)	33 (30.56)	
Marital status (%)			0.032			0.006			0.712
Living with partner/married	2578 (59.40)	197 (65.67)		1454 (63.52)	136 (73.51)		1124 (54.80)	61 (53.04)	
Never married/widowed/separated/divorced	1762 (40.60)	103 (34.33)		835 (36.48)	49 (26.49)		927 (45.20)	54 (46.96)	
Body mass index (%)			<0.001			<0.001			0.009
18.5 to <25 kg/m^2^	1371 (30.56)	43 (14.63)		688 (28.97)	21 (11.67)		683 (32.35)	22 (19.30)	
<18.5 kg/m^2^	83 (1.85)	2 (0.68)		37 (1.56)	0 (0.00)		46 (2.18)	2 (1.75)	
25 to <30 kg/m^2^	1499 (33.42)	82 (27.89)		896 (37.73)	50 (27.78)		603 (28.56)	32 (28.07)	
≥30 kg/m^2^	1533 (34.17)	167 (56.80)		754 (31.75)	109 (60.56)		779 (36.90)	58 (50.88)	
Physical activity			0.047			0.017			0.234
Moderate	1267 (28.26)	88 (29.53)		573 (24.17)	54 (29.67)		694 (32.84)	34 (29.31)	
Vigorous	1720 (38.36)	94 (31.54)		1118 (47.15)	66 (36.26)		602 (28.49)	28 (24.14)	
Other	1497 (33.39)	116 (38.93)		680 (28.68)	62 (34.07)		817 (38.67)	54 (46.55)	
Smoked at least 100 cigarettes in life (%)	1948 (46.47)	163 (54.70)	0.006	1222 (55.37)	115 (62.84)	0.050	726 (36.57)	48 (41.74)	0.264
Had at least 12 alcohol drinks/year (%)	2810 (73.52)	196 (71.01)	0.363	1735 (84.63)	140 (82.35)	0.430	1075 (60.67)	56 (52.83)	0.109
Use of female hormones (%)	247 (5.94)	27 (9.28)	0.022	-	-	-	247 (14.00)	27 (25.71)	0.001
Hypertension (%)	1973 (44.91)	188 (63.09)	<0.001	1154 (49.34)	128 (70.33)	<0.001	819 (39.87)	60 (51.72)	0.011
Depressive symptoms (%)	240 (5.86)	57 (20.58)	<0.001	78 (3.54)	23 (13.29)	<0.001	162 (8.56)	34 (32.69)	<0.001
Diabetes (%)	432 (9.70)	60 (20.98)	<0.001	209 (8.89)	39 (22.29)	<0.001	223 (10.60)	21 (18.92)	0.006
C-reactive protein (mg/dL), median (interquartile range)	0.17 (0.36)	0.24 (0.44)	<0.001	0.14 (0.27)	0.25 (0.41)	<0.001	0.21 (0.44)	0.22 (0.51)	0.225
Caffeine intake (mg/day), median (interquartile range)	92.00 (173.00)	128.00 (212.13)	0.001	103.50 (186.50)	132.75 (219.88)	0.012	82.00 (150.75)	103.25 (208.38)	0.057

The *p*-values were derived from Mann–Whitney *U* tests for non-normal continuous variables and chi-square tests for categorical variables.

**Table 2:** Weighted odds ratios (95% confidence intervals) for sleep disorders across tertiles of urinary phytoestrogens concentrations (National Health and Nutrition Examination Survey 2005–2010).

Phytoestrogen Concentrations (μg/g Creatinine)	Cases/Participants	Model 1 ^a^	Model 2 ^b^	Model 3 ^c^
Enterolactone				
Tertile 1 (<160.53)	107/1612	1.00 (reference)	1.00 (reference)	1.00 (reference)
Tertile 2 (160.53 to <621.68)	96/1609	0.83 (0.54–1.25)	0.78 (0.52–1.18)	0.99 (0.63–1.56)
Tertile 3 (≥621.68)	99/1609	0.64 (0.42–0.96) *	0.57 (0.38–0.85) **	0.64 (0.41–1.00) *
Enterodiol				
Tertile 1 (<21.52)	91/1611	1.00 (reference)	1.00 (reference)	1.00 (reference)
Tertile 2 (21.52 to <70.27)	103/1609	1.21 (0.88–1.65)	1.18 (0.86–1.62)	1.28 (0.86–1.91)
Tertile 3 (≥70.27)	108/1610	1.34 (0.88–2.05)	1.35 (0.89–2.07)	1.54 (1.07–2.21) *
Daidzein				
Tertile 1 (<24.22)	83/1611	1.00 (reference)	1.00 (reference)	1.00 (reference)
Tertile 2 (24.22 to <108.62)	106/1609	1.46 (0.82–2.61)	1.48 (0.83–2.63)	1.18 (0.65–2.15)
Tertile 3 (≥108.62)	113/1610	1.30 (0.61–2.79)	1.32 (0.62–2.81)	1.12 (0.54–2.30)
O-Desmethylangolensin				
Tertile 1 (<1.04)	84/1624	1.00 (reference)	1.00 (reference)	1.00 (reference)
Tertile 2 (1.04 to <9.06)	102/1613	1.33 (0.88–2.02)	1.32 (0.86–2.03)	1.38 (0.84–2.27)
Tertile 3 (≥9.06)	116/1593	1.65 (1.05–2.61) *	1.68 (1.06–2.65) *	1.89 (1.26–2.85) **
Equol				
Tertile 1 (<3.75)	84/1613	1.00 (reference)	1.00 (reference)	1.00 (reference)
Tertile 2 (3.75 to <9.73)	119/1612	1.21 (0.83–1.74)	1.16 (0.79–1.71)	1.01 (0.66–1.56)
Tertile 3 (≥9.73)	99/1605	0.86 (0.57–1.30)	0.84 (0.55–1.28)	0.76 (0.52–1.12)
Genistein				
Tertile 1 (<12.35)	104/1626	1.00 (reference)	1.00 (reference)	1.00 (reference)
Tertile 2 (12.35 to <50.00)	85/1601	0.60 (0.40–0.91) *	0.58 (0.38–0.88) *	0.68 (0.41–1.12)
Tertile 3 (≥50.00)	113/1603	0.80 (0.46–1.38)	0.74 (0.42–1.29)	0.78 (0.41–1.46)

^a^ Six phytoestrogens (tertiles) were entered into model 1 simultaneously. ^b^ Model 2 additionally adjusted for age and gender. ^c^ Model 3 further adjusted for race, education, marital status, occupation, family income, body mass index, physical activity, alcohol use, smoking status, depressive symptoms, diabetes, hypertension, use of female hormones, C-reactive protein (mg/dL), and caffeine intake (mg/day). * *p* < 0.05, ** *p* < 0.01.

**Table 3:** Weighted odds ratios (95% confidence intervals) for sleep disorders across tertiles of urinary phytoestrogens concentrations stratified by age (National Health and Nutrition Examination Survey 2005–2010).

Phytoestrogens Concentrations (μg/g Creatinine)	Cases/Participants	Model 1 ^a^	Model 2 ^b^	Model 3 ^c^
Age (18–39 years)				
Enterolactone				
Tertile 1 (<160.53)	33/767	1.00 (reference)	1.00 (reference)	1.00 (reference)
Tertile 2 (160.53 to <621.68)	23/683	0.60 (0.26–1.38)	0.60 (0.26–1.38)	0.86 (0.36–2.02)
Tertile 3 (≥621.68)	15/467	0.40 (0.16–0.97) *	0.40 (0.16–1.01)	0.65 (0.27–1.56)
Enterodiol				
Tertile 1 (<21.52)	28/732	1.00 (reference)	1.00 (reference)	1.00 (reference)
Tertile 2 (21.52 to <70.27)	24/620	1.55 (0.79–3.03)	1.57 (0.81–3.05)	1.62 (0.73–3.57)
Tertile 3 (≥70.27)	19/565	1.35 (0.63–2.92)	1.39 (0.64–3.00)	1.21 (0.46–3.15)
Daidzein				
Tertile 1 (<24.22)	16/679	1.00 (reference)	1.00 (reference)	1.00 (reference)
Tertile 2 (24.22 to <108.62)	32/661	1.88 (0.77–4.57)	1.89 (0.78–4.58)	1.17 (0.58–2.36)
Tertile 3 (≥108.62)	23/577	0.86 (0.30–2.49)	0.87 (0.31–2.47)	0.88 (0.18–4.30)
O-Desmethylangolensin				
Tertile 1 (<1.04)	19/707	1.00 (reference)	1.00 (reference)	1.00 (reference)
Tertile 2 (1.04 to <9.06)	21/621	1.23 (0.59–2.58)	1.24 (0.59–2.60)	1.34 (0.68–2.66)
Tertile 3 (≥9.06)	31/589	3.27 (1.39–7.70) **	3.28 (1.40–7.68) **	4.16 (1.58–10.97) **
Equol				
Tertile 1 (<3.75)	27/694	1.00 (reference)	1.00 (reference)	1.00 (reference)
Tertile 2 (3.75 to <9.73)	22/631	0.89 (0.41–1.94)	0.89 (0.42–1.93)	0.78 (0.36–1.70)
Tertile 3 (≥9.73)	22/592	0.72 (0.32–1.64)	0.73 (0.32–1.65)	0.44 (0.16–1.15)
Genistein				
Tertile 1 (<12.35)	19/701	1.00 (reference)	1.00 (reference)	1.00 (reference)
Tertile 2 (12.35 to <50.00)	30/650	1.08 (0.52–2.24)	1.08 (0.52–2.24)	1.18 (0.45–3.09)
Tertile 3 (≥50.00)	22/566	1.20 (0.49–2.93)	1.20 (0.49–2.94)	1.29 (0.31–5.39)
Age (40–59 years)				
Enterolactone				
Tertile 1 (<160.53)	39/533	1.00 (reference)	1.00 (reference)	1.00 (reference)
Tertile 2 (160.53 to <621.68)	37/468	0.89 (0.48–1.66)	0.89 (0.48–1.64)	1.00 (0.54–1.83)
Tertile 3 (≥ 621.68)	33/486	0.63 (0.31–1.29)	0.67 (0.33–1.38)	0.58 (0.28–1.22)
Enterodiol				
Tertile 1 (<21.52)	30/470	1.00 (reference)	1.00 (reference)	1.00 (reference)
Tertile 2 (21.52 to <70.27)	37/515	0.97 (0.53–1.75)	1.00 (0.56–1.78)	1.33 (0.62–2.87)
Tertile 3 (≥70.27)	42/502	1.29 (0.69–2.40)	1.36 (0.73–2.53)	2.56 (1.12–5.84) *
Daidzein				
Tertile 1 (<24.22)	36/521	1.00 (reference)	1.00 (reference)	1.00 (reference)
Tertile 2 (24.22 to <108.62)	30/469	1.20 (0.49–2.94)	1.14 (0.47–2.79)	1.37 (0.46–4.05)
Tertile 3 (≥108.62)	43/497	1.37 (0.43–4.34)	1.32 (0.42–4.19)	1.22 (0.31–4.74)
O-Desmethylangolensin				
Tertile 1 (<1.04)	31/522	1.00 (reference)	1.00 (reference)	1.00 (reference)
Tertile 2 (1.04 to <9.06)	35/481	1.84 (0.89–3.80)	1.86 (0.90–3.83)	1.65 (0.64–4.27)
Tertile 3 (≥9.06)	43/484	1.90 (0.92–3.93)	2.00 (0.98–4.05)	1.92 (0.78–4.76)
Equol				
Tertile 1 (<3.75)	32/534	1.00 (reference)	1.00 (reference)	1.00 (reference)
Tertile 2 (3.75 to <9.73)	40/487	1.17 (0.62–2.19)	1.20 (0.64–2.26)	1.07 (0.46–2.51)
Tertile 3 (≥9.73)	37/466	0.90 (0.48–1.70)	0.95 (0.50–1.80)	1.07 (0.51–2.24)
Genistein				
Tertile 1 (<12.35)	47/526	1.00 (reference)	1.00 (reference)	1.00 (reference)
Tertile 2 (12.35 to <50.00)	19/474	0.35 (0.16–0.77) *	0.34 (0.15–0.78) *	0.34 (0.13–0.88) *
Tertile 3 (≥50.00)	43/487	0.65 (0.21–1.97)	0.65 (0.21–2.00)	0.50 (0.17–1.48)
Age (≥60 years)				
Enterolactone				
Tertile 1 (<160.53)	35/312	1.00 (reference)	1.00 (reference)	1.00 (reference)
Tertile 2 (160.53 to <621.68)	36/458	0.87 (0.46–1.63)	0.80 (0.42–1.53)	1.21 (0.53–2.77)
Tertile 3 (≥621.68)	51/656	0.60 (0.34–1.06)	0.58 (0.32–1.03)	0.84 (0.38–1.90)
Enterodiol				
Tertile 1 (<21.52)	33/409	1.00 (reference)	1.00 (reference)	1.00 (reference)
Tertile 2 (21.52 to <70.27)	42/474	1.21 (0.67–2.16)	1.22 (0.68–2.20)	1.01 (0.52–1.96)
Tertile 3 (≥70.27)	47/543	1.26 (0.67–2.37)	1.34 (0.71–2.50)	0.88 (0.43–1.78)
Daidzein				
Tertile 1 (<24.22)	31/411	1.00 (reference)	1.00 (reference)	1.00 (reference)
Tertile 2 (24.22 to <108.62)	44/479	1.93 (0.85–4.36)	1.88 (0.82–4.29)	1.27 (0.48–3.35)
Tertile 3 (≥108.62)	47/536	2.36 (0.65–8.53)	2.26 (0.63–8.16)	1.59 (0.43–5.93)
O-Desmethylangolensin				
Tertile 1 (<1.04)	34/395	1.00 (reference)	1.00 (reference)	1.00 (reference)
Tertile 2 (1.04 to <9.06)	46/511	0.73 (0.38–1.38)	0.75 (0.39–1.44)	0.78 (0.33–1.80)
Tertile 3 (≥9.06)	42/520	0.66 (0.34–1.29)	0.70 (0.35–1.39)	0.87 (0.34–2.28)
Equol				
Tertile 1 (<3.75)	25/385	1.00 (reference)	1.00 (reference)	1.00 (reference)
Tertile 2 (3.75 to <9.73)	57/494	1.41 (0.79–2.54)	1.41 (0.78–2.55)	1.01 (0.42–2.45)
Tertile 3 (≥9.73)	40/547	0.75 (0.36–1.56)	0.76 (0.37–1.59)	0.56 (0.25–1.24)
Genistein				
Tertile 1 (<12.35)	38/399	1.00 (reference)	1.00 (reference)	1.00 (reference)
Tertile 2 (12.35 to <50.00)	36/477	0.60 (0.29–1.26)	0.60 (0.29–1.25)	0.77 (0.29–2.06)
Tertile 3 (≥50.00)	48/550	0.51 (0.16–1.61)	0.53 (0.17–1.64)	0.86 (0.19–3.85)

^a^ Six phytoestrogens (tertiles) were entered into model 1 simultaneously. ^b^ Model 2 additionally adjusted for gender. ^c^ Model 3 further adjusted for race, education, marital status, occupation, family income, body mass index, physical activity, alcohol use, smoking status, depressive symptoms, diabetes, hypertension, use of female hormones, C-reactive protein (mg/dL), and caffeine intake (mg/day). * *p* < 0.05, ** *p* < 0.01.

**Table 4:** Weighted odds ratios (95% confidence intervals) for sleep disorders across tertiles of urinary phytoestrogen concentrations stratified by gender (National Health and Nutrition Examination Survey 2005–2010).

Phytoestrogen Concentrations (μg/g Creatinine)	Cases/Participants	Model 1 ^a^	Model 2 ^b^	Model 3 ^c^
Females				
Enterolactone				
Tertile 1 (<160.53)	40/698	1.00 (reference)	1.00 (reference)	1.00 (reference)
Tertile 2 (160.53 to <621.68)	36/706	0.52 (0.24–1.14)	0.53 (0.24–1.16)	0.56 (0.25–1.26)
Tertile 3 (≥621.68)	40/844	0.36 (0.19–0.68) **	0.32 (0.17–0.61) **	0.45 (0.21–0.94) *
Enterodiol				
Tertile 1 (<21.52)	28/649	1.00 (reference)	1.00 (reference)	1.00 (reference)
Tertile 2 (21.52 to <70.27)	33/728	1.90 (1.04–3.48) *	1.81 (0.98–3.32)	2.70 (1.60–4.55) ***
Tertile 3 (≥70.27)	55/871	2.89 (1.54–5.42) **	2.79 (1.46–5.31) **	3.79 (1.83–7.87) **
Daidzein				
Tertile 1 (<24.22)	21/708	1.00 (reference)	1.00 (reference)	1.00 (reference)
Tertile 2 (24.22 to <108.62)	51/745	2.16 (0.93–5.00)	2.33 (1.01–5.39) *	1.82 (0.77–4.30)
Tertile 3 (≥108.62)	44/795	1.71 (0.56–5.20)	1.82 (0.59–5.55)	1.44 (0.48–4.28)
O-Desmethylangolensin				
Tertile 1 (<1.04)	27/697	1.00 (reference)	1.00 (reference)	1.00 (reference)
Tertile 2 (1.04 to <9.06)	41/753	1.70 (0.82–3.52)	1.68 (0.79–3.57)	2.04 (0.95–4.37)
Tertile 3 (≥9.06)	48/798	2.00 (1.00–3.99)	1.94 (1.00–3.76)	2.09 (0.96–4.56)
Equol				
Tertile 1 (<3.75)	31/669	1.00 (reference)	1.00 (reference)	1.00 (reference)
Tertile 2 (3.75 to <9.73)	47/752	1.36 (0.74–2.48)	1.30 (0.71–2.37)	1.25 (0.57–2.73)
Tertile 3 (≥9.73)	38/827	0.85 (0.41–1.78)	0.80 (0.38–1.68)	0.62 (0.28–1.35)
Genistein				
Tertile 1 (<12.35)	33/720	1.00 (reference)	1.00 (reference)	1.00 (reference)
Tertile 2 (12.35 to <50.00)	39/723	0.60 (0.31–1.17)	0.57 (0.29–1.12)	0.59 (0.25–1.38)
Tertile 3 (≥50.00)	44/805	0.62 (0.27–1.42)	0.57 (0.25–1.30)	0.61 (0.23–1.57)
Males				
Enterolactone				
Tertile 1 (<160.53)	67/914	1.00 (reference)	1.00 (reference)	1.00 (reference)
Tertile 2 (160.53 to <621.68)	60/903	1.16 (0.67–1.99)	1.08 (0.63–1.85)	1.58 (0.86–2.91)
Tertile 3 (≥621.68)	59/765	1.11 (0.65–1.88)	0.93 (0.54–1.61)	0.92 (0.46–1.83)
Enterodiol				
Tertile 1 (<21.52)	63/962	1.00 (reference)	1.00 (reference)	1.00 (reference)
Tertile 2 (21.52 to <70.27)	70/881	0.99 (0.64–1.52)	0.94 (0.61–1.46)	0.92 (0.51–1.65)
Tertile 3 (≥70.27)	53/739	0.88 (0.52–1.50)	0.85 (0.51–1.42)	0.92 (0.56–1.53)
Daidzein				
Tertile 1 (<24.22)	62/903	1.00 (reference)	1.00 (reference)	1.00 (reference)
Tertile 2 (24.22 to <108.62)	55/864	1.10 (0.58–2.06)	1.07 (0.58–1.98)	0.88 (0.48–1.64)
Tertile 3 (≥108.62)	69/815	1.02 (0.41–2.52)	1.01 (0.42–2.45)	1.05 (0.44–2.50)
O-Desmethylangolensin				
Tertile 1 (<1.04)	57/927	1.00 (reference)	1.00 (reference)	1.00 (reference)
Tertile 2 (1.04 to <9.06)	61/860	1.17 (0.65–2.11)	1.15 (0.65–2.04)	1.05 (0.53–2.05)
Tertile 3 (≥9.06)	68/795	1.57 (0.88–2.80)	1.58 (0.89–2.80)	1.58 (0.89–2.79)
Equol				
Tertile 1 (<3.75)	53/944	1.00 (reference)	1.00 (reference)	1.00 (reference)
Tertile 2 (3.75 to <9.73)	72/860	1.12 (0.70–1.81)	1.06 (0.65–1.73)	0.79 (0.47–1.35)
Tertile 3 (≥9.73)	61/778	0.91 (0.54–1.54)	0.88 (0.52–1.48)	0.86 (0.47–1.58)
Genistein				
Tertile 1 (<12.35)	71/906	1.00 (reference)	1.00 (reference)	1.00 (reference)
Tertile 2 (12.35 to <50.00)	46/878	0.59 (0.36–0.97) *	0.57 (0.35–0.93) *	0.74 (0.41–1.33)
Tertile 3 (≥50.00)	69/798	1.04 (0.52–2.10)	0.96 (0.48–1.91)	0.96 (0.42–2.20)

^a^ Six phytoestrogens (tertiles) were entered into model 1 simultaneously. ^b^ Model 2 additionally adjusted for age. ^c^ Model 3 further adjusted for race, education, marital status, occupation, family income, body mass index, physical activity, alcohol use, smoking status, depressive symptoms, diabetes, hypertension, use of female hormones (only in females), C-reactive protein (mg/dL), and caffeine intake (mg/day). * *p* < 0.05, ** *p* < 0.01, *** *p* < 0.001.

**Table 5:** Weighted odds ratios (95% confidence intervals) for sleep disorders across tertiles of urinary phytoestrogen concentrations stratified by age for males and females (National Health and Nutrition Examination Survey 2005–2010).

Phytoestrogen Concentrations (μg/g Creatinine)	Males		Females	
	Cases/Participants	Model 3 ^a^	Cases/Participants	Model 3 ^a^
Age (18–39 years)				
Enterolactone				
Tertile 1 (<160.53)	18/444	1.00 (reference)	15/323	1.00 (reference)
Tertile 2 (160.53 to <621.68)	13/353	1.65 (0.53–5.08)	10/330	0.54 (0.14–2.14)
Tertile 3 (≥621.68)	9/200	1.12 (0.27–4.69)	6/267	0.46 (0.10–2.14)
Enterodiol				
Tertile 1 (<21.52)	19/434	1.00 (reference)	9/298	1.00 (reference)
Tertile 2 (21.52 to <70.27)	12/328	0.90 (0.28–2.89)	12/292	6.52 (1.69–25.23) **
Tertile 3 (≥70.27)	9/235	1.28 (0.36–4.59)	10/330	1.84 (0.50–6.80)
Daidzein				
Tertile 1 (<24.22)	12/381	1.00 (reference)	4/298	1.00 (reference)
Tertile 2 (24.22 to <108.62)	16/327	0.86 (0.38–1.93)	16/334	2.35 (0.60–9.22)
Tertile 3 (≥108.62)	12/289	0.45 (0.09–2.25)	11/288	5.07 (0.52–49.84)
O-Desmethylangolensin				
Tertile 1 (<1.04)	10/395	1.00 (reference)	9/312	1.00 (reference)
Tertile 2 (1.04 to <9.06)	12/311	1.28 (0.39–4.18)	9/310	1.18 (0.37–3.72)
Tertile 3 (≥9.06)	18/291	6.57 (2.06–20.99) **	13/298	0.74 (0.12–4.45)
Equol				
Tertile 1 (<3.75)	15/396	1.00 (reference)	12/298	1.00 (reference)
Tertile 2 (3.75 to <9.73)	9/318	0.44 (0.15–1.33)	13/313	1.37 (0.38–4.88)
Tertile 3 (≥9.73)	16/283	0.74 (0.29–1.86)	6/309	0.08 (0.01–0.84) *
Genistein				
Tertile 1 (<12.35)	13/386	1.00 (reference)	6/315	1.00 (reference)
Tertile 2 (12.35 to <50.00)	15/337	1.60 (0.54–4.73)	15/313	1.10 (0.29–4.10)
Tertile 3 (≥50.00)	12/274	1.70 (0.37–7.85)	10/292	0.59 (0.12–2.81)
Age (40–59 years)				
Enterolactone				
Tertile 1 (<160.53)	25/304	1.00 (reference)	14/229	1.00 (reference)
Tertile 2 (160.53 to <621.68)	24/260	1.90 (0.74–4.88)	13/208	0.50 (0.16–1.53)
Tertile 3 (≥621.68)	17/209	1.15 (0.36–3.61)	16/277	0.32 (0.10–1.03)
Enterodiol				
Tertile 1 (<21.52)	23/272	1.00 (reference)	7/198	1.00 (reference)
Tertile 2 (21.52 to <70.27)	24/272	1.04 (0.32–3.42)	13/243	3.02 (1.10–8.30) *
Tertile 3 (≥70.27)	19/229	1.47 (0.43–5.00)	23/273	13.66 (2.06–90.70) **
Daidzein				
Tertile 1 (<24.22)	25/281	1.00 (reference)	11/240	1.00 (reference)
Tertile 2 (24.22 to <108.62)	15/262	2.04 (0.45–9.16)	15/207	1.65 (0.41–6.72)
Tertile 3 (≥108.62)	26/230	2.94 (0.38–22.73)	17/267	0.85 (0.13–5.75)
O-Desmethylangolensin				
Tertile 1 (<1.04)	25/297	1.00 (reference)	6/225	1.00 (reference)
Tertile 2 (1.04 to <9.06)	18/252	0.56 (0.18–1.79)	17/229	11.50 (2.14–61.72) **
Tertile 3 (≥9.06)	23/224	0.68 (0.21–2.20)	20/260	15.14 (2.99–76.65) **
Equol				
Tertile 1 (<3.75)	21/316	1.00 (reference)	11/218	1.00 (reference)
Tertile 2 (3.75 to <9.73)	27/251	0.88 (0.27–2.86)	13/236	0.95 (0.29–3.12)
Tertile 3 (≥9.73)	18/206	1.28 (0.43–3.80)	19/260	0.77 (0.26–2.31)
Genistein				
Tertile 1 (<12.35)	30/285	1.00 (reference)	17/241	1.00 (reference)
Tertile 2 (12.35 to <50.00)	10/256	0.29 (0.05–1.61)	9/218	0.20 (0.04–1.00)
Tertile 3 (≥50.00)	26/232	0.43 (0.06–3.03)	17/255	0.38 (0.07–2.05)
Age (≥60 years)				
Enterolactone				
Tertile 1 (<160.53)	24/166	1.00 (reference)	11/146	1.00 (reference)
Tertile 2 (160.53 to <621.68)	23/290	1.59 (0.63–4.04)	13/168	1.37 (0.43–4.40)
Tertile 3 (≥621.68)	33/356	0.77 (0.34–1.75)	18/300	1.47 (0.29–7.40)
Enterodiol				
Tertile 1 (<21.52)	21/256	1.00 (reference)	12/153	1.00 (reference)
Tertile 2 (21.52 to <70.27)	34/281	0.98 (0.44–2.20)	8/193	1.18 (0.26–5.28)
Tertile 3 (≥70.27)	25/275	0.48 (0.20–1.16)	22/268	2.00 (0.71–5.57)
Daidzein				
Tertile 1 (<24.22)	25/241	1.00 (reference)	6/170	1.00 (reference)
Tertile 2 (24.22 to <108.62)	24/275	0.47 (0.16–1.39)	20/204	13.45 (2.89–62.50) **
Tertile 3 (≥108.62)	31/296	0.80 (0.13–4.79)	16/240	10.67 (1.88–60.44) **
O-Desmethylangolensin				
Tertile 1 (<1.04)	22/235	1.00 (reference)	12/160	1.00 (reference)
Tertile 2 (1.04 to <9.06)	31/297	1.16 (0.37–3.66)	15/214	0.27 (0.06–1.19)
Tertile 3 (≥9.06)	27/280	1.19 (0.33–4.37)	15/240	0.25 (0.10–0.58) **
Equol				
Tertile 1 (<3.75)	17/232	1.00 (reference)	8/153	1.00 (reference)
Tertile 2 (3.75 to <9.73)	36/291	0.69 (0.23–2.08)	21/203	2.05 (0.63–6.68)
Tertile 3 (≥9.73)	27/289	0.72 (0.28–1.83)	13/258	0.49 (0.12–1.97)
Genistein				
Tertile 1 (<12.35)	28/235	1.00 (reference)	10/164	1.00 (reference)
Tertile 2 (12.35 to <50.00)	21/285	0.75 (0.22–2.53)	15/192	0.71 (0.14–3.53)
Tertile 3 (≥50.00)	31/292	1.29 (0.16–10.26)	17/258	0.41 (0.06–2.82)

^a^ Adjusted for education, marital status, occupation, family income, body mass index, physical activity, alcohol use, smoking status, depressive symptoms, diabetes, hypertension, use of female hormones (only in females), C-reactive protein (mg/dL), caffeine intake (mg/day), and the other five phytoestrogens (tertiles). * *p* < 0.05, ** *p* < 0.01.

**Table 6:** Weighted odds ratios (95% confidence intervals) for sleep disorders across tertiles of urinary phytoestrogen concentrations stratified by race (National Health and Nutrition Examination Survey 2005–2010).

Phytoestrogen Concentrations (μg/g Creatinine)	Cases/Participants	Model 3 ^a^
Mexican American		
Enterolactone		
Tertile 1 (<160.53)	9/298	1.00 (reference)
Tertile 2 (160.53 to <621.68)	11/347	2.24 (0.28–17.56)
Tertile 3 (≥621.68)	19/308	2.47 (0.54–11.42)
Enterodiol		
Tertile 1 (<21.52)	9/366	1.00 (reference)
Tertile 2 (21.52 to <70.27)	15/315	1.64 (0.85–3.17)
Tertile 3 (≥70.27)	15/272	1.91 (0.56–6.50)
Daidzein		
Tertile 1 (<24.22)	10/359	1.00 (reference)
Tertile 2 (24.22 to <108.62)	12/292	0.72 (0.14–3.83)
Tertile 3 (≥108.62)	17/302	1.03 (0.11–9.46)
O-Desmethylangolensin		
Tertile 1 (<1.04)	7/401	1.00 (reference)
Tertile 2 (1.04 to <9.06)	18/312	2.00 (0.53–7.61)
Tertile 3 (≥9.06)	14/240	2.16 (0.73–6.39)
Equol		
Tertile 1 (<3.75)	13/421	1.00 (reference)
Tertile 2 (3.75 to <9.73)	17/331	0.64 (0.17–2.45)
Tertile 3 (≥9.73)	9/201	0.37 (0.09–1.48)
Genistein		
Tertile 1 (<12.35)	10/341	1.00 (reference)
Tertile 2 (12.35 to <50.00)	13/314	2.26 (0.35–14.60)
Tertile 3 (≥50.00)	16/298	2.00 (0.14–28.91)
Non-Hispanic White		
Enterolactone		
Tertile 1 (<160.53)	57/673	1.00 (reference)
Tertile 2 (160.53 to <621.68)	59/681	1.00 (0.60–1.67)
Tertile 3 (≥621.68)	57/859	0.56 (0.33–0.95) *
Enterodiol		
Tertile 1 (<21.52)	43/606	1.00 (reference)
Tertile 2 (21.52 to <70.27)	62/765	1.52 (0.88–2.63)
Tertile 3 (≥70.27)	68/842	1.89 (1.13–3.13) *
Daidzein		
Tertile 1 (<24.22)	45/684	1.00 (reference)
Tertile 2 (24.22 to <108.62)	62/771	1.12 (0.55–2.29)
Tertile 3 (≥108.62)	66/758	0.95 (0.41–2.21)
O-Desmethylangolensin		
Tertile 1 (<1.04)	44/638	1.00 (reference)
Tertile 2 (1.04 to <9.06)	60/798	1.48 (0.76–2.90)
Tertile 3 (≥9.06)	69/777	2.16 (1.31–3.55) **
Equol		
Tertile 1 (<3.75)	39/493	1.00 (reference)
Tertile 2 (3.75 to <9.73)	67/729	0.97 (0.57–1.64)
Tertile 3 (≥9.73)	67/991	0.76 (0.47–1.22)
Genistein		
Tertile 1 (<12.35)	60/696	1.00 (reference)
Tertile 2 (12.35 to <50.00)	48/766	0.63 (0.34–1.16)
Tertile 3 (≥50.00)	65/751	0.86 (0.42–1.76)
Non-Hispanic Black		
Enterolactone		
Tertile 1 (<160.53)	20/388	1.00 (reference)
Tertile 2 (160.53 to <621.68)	17/385	1.11 (0.48–2.55)
Tertile 3 (≥621.68)	17/256	0.86 (0.26–2.89)
Enterodiol		
Tertile 1 (<21.52)	23/423	1.00 (reference)
Tertile 2 (21.52 to <70.27)	16/338	0.81 (0.29–2.25)
Tertile 3 (≥70.27)	15/268	0.79 (0.40–1.57)
Daidzein		
Tertile 1 (<24.22)	16/371	1.00 (reference)
Tertile 2 (24.22 to <108.62)	24/331	2.05 (0.57–7.36)
Tertile 3 (≥108.62)	14/327	0.91 (0.16–5.32)
O-Desmethylangolensin		
Tertile 1 (<1.04)	15/338	1.00 (reference)
Tertile 2 (1.04 to <9.06)	17/331	2.53 (0.86–7.50)
Tertile 3 (≥9.06)	22/360	2.23 (0.49–10.14)
Equol		
Tertile 1 (<3.75)	20/486	1.00 (reference)
Tertile 2 (3.75 to <9.73)	23/327	0.99 (0.44–2.26)
Tertile 3 (≥9.73)	11/216	0.54 (0.17–1.75)
Genistein		
Tertile 1 (<12.35)	24/411	1.00 (reference)
Tertile 2 (12.35 to <50.00)	16/313	0.51 (0.20–1.28)
Tertile 3 (≥50.00)	14/305	0.73 (0.18–3.02)
Other Hispanic		
Enterolactone		
Tertile 1 (<160.53)	16/159	1.00 (reference)
Tertile 2 (160.53 to <621.68)	8/135	1.34 (0.38–4.75)
Tertile 3 (≥621.68)	4/119	0.45 (0.03–6.95)
Enterodiol		
Tertile 1 (<21.52)	13/159	1.00 (reference)
Tertile 2 (21.52 to <70.27)	6/123	0.25 (0.05–1.31)
Tertile 3 (≥70.27)	9/131	2.76 (0.49–15.59)
Daidzein		
Tertile 1 (<24.22)	9/147	1.00 (reference)
Tertile 2 (24.22 to <108.62)	7/141	1.43 (0.21–9.83)
Tertile 3 (≥108.62)	12/125	18.97 (0.50–719.28)
O-Desmethylangolensin		
Tertile 1 (<1.04)	15/171	1.00 (reference)
Tertile 2 (1.04 to <9.06)	6/120	0.13 (0.02–0.86) *
Tertile 3 (≥9.06)	7/122	0.10 (0.01–1.32)
Equol		
Tertile 1 (<3.75)	8/130	1.00 (reference)
Tertile 2 (3.75 to <9.73)	8/137	0.46 (0.13–1.70)
Tertile 3 (≥9.73)	12/146	3.22 (1.11–9.30) *
Genistein		
Tertile 1 (<12.35)	7/132	1.00 (reference)
Tertile 2 (12.35 to <50.00)	6/144	0.58 (0.12–2.72)
Tertile 3 (≥50.00)	15/137	0.30 (0.03–2.91)

^a^ Adjusted for age, gender, education, marital status, occupation, family income, body mass index, physical activity, alcohol use, smoking status, depressive symptoms, diabetes, hypertension, use of female hormones, C-reactive protein (mg/dL), caffeine intake (mg/day), and the other five phytoestrogens (tertiles). * *p* < 0.05, ** *p* < 0.01.

**Table 7:** Weighted relative risk ratios (95% confidence intervals) for sleep duration (reference, 7–8 h/night) across tertiles of the urinary phytoestrogen concentrations (National Health and Nutrition Examination Survey 2005–2010).

Phytoestrogen Concentrations (μg/g Creatinine)	Model 3 ^a^		
	Very Short Sleep (<5 h/Night)	Short Sleep (5–6 h/Night)	Long Sleep (≥9 h/Night)
Enterolactone			
Tertile 1 (<160.53)	1.00 (reference)	1.00 (reference)	1.00 (reference)
Tertile 2 (160.53 to <621.68)	0.98 (0.64–1.50)	1.15 (0.86–1.54)	1.46 (0.93–2.28)
Tertile 3 (≥621.68)	0.56 (0.36–0.86) **	0.94 (0.69–1.27)	0.78 (0.45–1.33)
Enterodiol			
Tertile 1 (<21.52)	1.00 (reference)	1.00 (reference)	1.00 (reference)
Tertile 2 (21.52 to <70.27)	1.35 (0.76–2.42)	1.09 (0.84–1.40)	1.04 (0.65–1.67)
Tertile 3 (≥70.27)	0.97 (0.54–1.76)	1.04 (0.77–1.39)	1.46 (0.88–2.40)
Daidzein			
Tertile 1 (<24.22)	1.00 (reference)	1.00 (reference)	1.00 (reference)
Tertile 2 (24.22 to <108.62)	1.02 (0.49–2.13)	1.08 (0.85–1.37)	1.10 (0.63–1.93)
Tertile 3 (≥108.62)	1.49 (0.48–4.63)	1.29 (0.83–2.03)	1.15 (0.54–2.43)
O-Desmethylangolensin			
Tertile 1 (<1.04)	1.00 (reference)	1.00 (reference)	1.00 (reference)
Tertile 2 (1.04 to <9.06)	1.43 (0.83–2.47)	1.01 (0.82–1.26)	1.05 (0.67–1.64)
Tertile 3 (≥9.06)	0.82 (0.36–1.88)	0.86 (0.61–1.21)	1.44 (0.80–2.56)
Equol			
Tertile 1 (<3.75)	1.00 (reference)	1.00 (reference)	1.00 (reference)
Tertile 2 (3.75 to <9.73)	1.01 (0.71–1.44)	1.04 (0.81–1.33)	1.06 (0.69–1.62)
Tertile 3 (≥9.73)	0.89 (0.50–1.58)	1.05 (0.84–1.31)	0.92 (0.60–1.41)
Genistein			
Tertile 1 (<12.35)	1.00 (reference)	1.00 (reference)	1.00 (reference)
Tertile 2 (12.35 to <50.00)	0.94 (0.47–1.89)	1.10 (0.85–1.43)	0.62 (0.39–0.99) *
Tertile 3 (≥50.00)	1.05 (0.44–2.53)	0.95 (0.65–1.40)	0.62 (0.33–1.15)

^a^ Adjusted for age, gender, race, education, marital status, occupation, family income, body mass index, physical activity, alcohol use, smoking status, depressive symptoms, diabetes, hypertension, use of female hormones, C-reactive protein (mg/dL), caffeine intake (mg/day), and the other five phytoestrogens (tertiles). * *p* < 0.05, ** *p* < 0.01.

**Table 8:** Weighted relative risk ratios (95% confidence intervals) for sleep duration (reference, 7–8 h/night) across tertiles of the urinary phytoestrogen concentrations stratified by age (National Health and Nutrition Examination Survey 2005–2010).

Phytoestrogen Concentrations (μg/g Creatinine)	Model 3 ^a^		
	Very Short Sleep (<5 h/Night)	Short Sleep (5–6 h/Night)	Long Sleep (≥9 h/Night)
Age (18–39 years)			
Enterolactone			
Tertile 1 (<160.53)	1.00 (reference)	1.00 (reference)	1.00 (reference)
Tertile 2 (160.53 to <621.68)	0.69 (0.30–1.59)	0.96 (0.62–1.48)	2.21 (0.99–4.90)
Tertile 3 (≥621.68)	0.24 (0.09–0.61) **	0.78 (0.45–1.36)	1.33 (0.53–3.33)
Enterodiol			
Tertile 1 (<21.52)	1.00 (reference)	1.00 (reference)	1.00 (reference)
Tertile 2 (21.52 to <70.27)	2.73 (1.20–6.21) *	1.20 (0.72–2.01)	0.72 (0.33–1.58)
Tertile 3 (≥70.27)	0.81 (0.25–2.63)	1.00 (0.59–1.69)	1.63 (0.71–3.77)
Daidzein			
Tertile 1 (<24.22)	1.00 (reference)	1.00 (reference)	1.00 (reference)
Tertile 2 (24.22 to <108.62)	1.24 (0.28–5.41)	1.05 (0.72–1.52)	0.58 (0.19–1.73)
Tertile 3 (≥108.62)	1.94 (0.19–19.60)	1.34 (0.65–2.74)	1.09 (0.35–3.36)
O-Desmethylangolensin			
Tertile 1 (<1.04)	1.00 (reference)	1.00 (reference)	1.00 (reference)
Tertile 2 (1.04 to <9.06)	1.75 (0.54–5.73)	1.03 (0.69–1.53)	0.64 (0.27–1.52)
Tertile 3 (≥9.06)	1.21 (0.24–6.20)	0.84 (0.52–1.34)	1.28 (0.54–3.08)
Equol			
Tertile 1 (<3.75)	1.00 (reference)	1.00 (reference)	1.00 (reference)
Tertile 2 (3.75 to <9.73)	1.67 (0.70–4.00)	1.09 (0.71–1.67)	0.88 (0.44–1.76)
Tertile 3 (≥9.73)	0.69 (0.23–2.03)	1.03 (0.67–1.59)	0.68 (0.32–1.45)
Genistein			
Tertile 1 (<12.35)	1.00 (reference)	1.00 (reference)	1.00 (reference)
Tertile 2 (12.35 to <50.00)	1.52 (0.46–5.00)	1.21 (0.79–1.83)	1.35 (0.59–3.08)
Tertile 3 (≥50.00)	1.61 (0.36–7.19)	0.91 (0.46–1.79)	0.92 (0.31–2.71)
Age (40-59 years)			
Enterolactone			
Tertile 1 (<160.53)	1.00 (reference)	1.00 (reference)	1.00 (reference)
Tertile 2 (160.53 to <621.68)	1.32 (0.70–2.49)	1.44 (0.87–2.38)	1.98 (0.98–4.03)
Tertile 3 (≥621.68)	0.87 (0.37–2.05)	1.25 (0.80–1.94)	0.55 (0.19–1.58)
Enterodiol			
Tertile 1 (<21.52)	1.00 (reference)	1.00 (reference)	1.00 (reference)
Tertile 2 (21.52 to <70.27)	0.95 (0.40–2.29)	0.97 (0.65–1.44)	0.69 (0.24–1.94)
Tertile 3 (≥70.27)	0.59 (0.23–1.55)	0.93 (0.65–1.33)	1.45 (0.69–3.07)
Daidzein			
Tertile 1 (<24.22)	1.00 (reference)	1.00 (reference)	1.00 (reference)
Tertile 2 (24.22 to <108.62)	0.77 (0.26–2.27)	1.12 (0.64–1.96)	2.42 (0.96–6.12)
Tertile 3 (≥108.62)	1.69 (0.39–7.36)	1.45 (0.62–3.42)	3.92 (0.55–27.81)
O-Desmethylangolensin			
Tertile 1 (<1.04)	1.00 (reference)	1.00 (reference)	1.00 (reference)
Tertile 2 (1.04 to <9.06)	1.37 (0.59–3.17)	0.87 (0.59–1.26)	1.54 (0.58–4.11)
Tertile 3 (≥9.06)	0.43 (0.12–1.60)	0.71 (0.35–1.44)	1.41 (0.56–3.58)
Equol			
Tertile 1 (<3.75)	1.00 (reference)	1.00 (reference)	1.00 (reference)
Tertile 2 (3.75 to <9.73)	0.41 (0.14–1.22)	0.98 (0.65–1.48)	1.42 (0.68–2.96)
Tertile 3 (≥9.73)	0.65 (0.25–1.73)	0.95 (0.67–1.36)	1.14 (0.37–3.55)
Genistein			
Tertile 1 (<12.35)	1.00 (reference)	1.00 (reference)	1.00 (reference)
Tertile 2 (12.35 to <50.00)	0.73 (0.27–2.01)	1.09 (0.68–1.76)	0.14 (0.05–0.40) ***
Tertile 3 (≥50.00)	0.85 (0.23–3.17)	1.00 (0.53–1.86)	0.10 (0.02–0.56) *
Age (≥60 years)			
Enterolactone			
Tertile 1 (<160.53)	1.00 (reference)	1.00 (reference)	1.00 (reference)
Tertile 2 (160.53 to <621.68)	1.75 (0.62–4.94)	1.07 (0.61–1.87)	0.61 (0.29–1.30)
Tertile 3 (≥621.68)	1.27 (0.52–3.10)	0.81 (0.52–1.27)	0.49 (0.29–0.84) *
Enterodiol			
Tertile 1 (<21.52)	1.00 (reference)	1.00 (reference)	1.00 (reference)
Tertile 2 (21.52 to <70.27)	0.45 (0.16–1.30)	1.09 (0.65–1.84)	1.56 (0.82–2.96)
Tertile 3 (≥70.27)	1.38 (0.63–3.02)	1.11 (0.66–1.88)	1.64 (0.92–2.93)
Daidzein			
Tertile 1 (<24.22)	1.00 (reference)	1.00 (reference)	1.00 (reference)
Tertile 2 (24.22 to <108.62)	0.94 (0.11–7.95)	1.07 (0.61–1.88)	1.35 (0.68–2.68)
Tertile 3 (≥108.62)	0.62 (0.04–10.32)	1.08 (0.46–2.55)	0.62 (0.26–1.48)
O-Desmethylangolensin			
Tertile 1 (<1.04)	1.00 (reference)	1.00 (reference)	1.00 (reference)
Tertile 2 (1.04 to <9.06)	1.21 (0.47–3.14)	1.53 (0.92–2.53)	1.33 (0.72–2.45)
Tertile 3 (≥9.06)	0.75 (0.17–3.22)	1.21 (0.64–2.27)	1.38 (0.66–2.89)
Equol			
Tertile 1 (<3.75)	1.00 (reference)	1.00 (reference)	1.00 (reference)
Tertile 2 (3.75 to <9.73)	1.26 (0.52–3.06)	0.95 (0.54–1.67)	1.07 (0.48–2.40)
Tertile 3 (≥9.73)	1.93 (0.81–4.62)	1.10 (0.66–1.86)	1.16 (0.59–2.26)
Genistein			
Tertile 1 (<12.35)	1.00 (reference)	1.00 (reference)	1.00 (reference)
Tertile 2 (12.35 to <50.00)	0.89 (0.14–5.68)	0.84 (0.47–1.51)	0.50 (0.23–1.09)
Tertile 3 (≥50.00)	1.77 (0.15–21.09)	0.73 (0.37–1.42)	1.32 (0.59–2.94)

^a^ Adjusted for gender, race, education, marital status, occupation, family income, body mass index, physical activity, alcohol use, smoking status, depressive symptoms, diabetes, hypertension, use of female hormones, C-reactive protein (mg/dL), caffeine intake (mg/day), and the other five phytoestrogens (tertiles). * *p* < 0.05, ** *p* < 0.01, *** *p* < 0.001.

**Table 9:** Weighted relative risk ratios (95% confidence intervals) for sleep duration (reference, 7–8 h/night) across tertiles of the urinary phytoestrogen concentrations stratified by gender (National Health and Nutrition Examination Survey 2005–2010).

Phytoestrogen Concentrations (μg/g Creatinine)	Model 3 ^a^		
	Very Short Sleep (<5 h/Night)	Short Sleep (5–6 h/Night)	Long Sleep (≥9 h/Night)
Females			
Enterolactone			
Tertile 1 (<160.53)	1.00 (reference)	1.00 (reference)	1.00 (reference)
Tertile 2 (160.53 to <621.68)	0.69 (0.38–1.27)	0.99 (0.63–1.55)	1.55 (0.83–2.87)
Tertile 3 (≥621.68)	0.61 (0.31–1.20)	0.91 (0.62–1.35)	0.74 (0.35–1.54)
Enterodiol			
Tertile 1 (<21.52)	1.00 (reference)	1.00 (reference)	1.00 (reference)
Tertile 2 (21.52 to <70.27)	1.39 (0.70–2.77)	0.77 (0.53–1.13)	1.03 (0.56–1.90)
Tertile 3 (≥70.27)	0.91 (0.42–1.99)	0.99 (0.61–1.59)	2.14 (1.11–4.13) *
Daidzein			
Tertile 1 (<24.22)	1.00 (reference)	1.00 (reference)	1.00 (reference)
Tertile 2 (24.22 to <108.62)	1.50 (0.58–3.89)	0.85 (0.57–1.28)	0.79 (0.34–1.86)
Tertile 3 (≥108.62)	1.69 (0.35–8.12)	0.72 (0.41–1.28)	0.75 (0.27–2.10)
O-Desmethylangolensin			
Tertile 1 (<1.04)	1.00 (reference)	1.00 (reference)	1.00 (reference)
Tertile 2 (1.04 to <9.06)	1.33 (0.63–2.80)	1.25 (0.83–1.87)	1.19 (0.64–2.20)
Tertile 3 (≥9.06)	0.87 (0.32–2.39)	1.23 (0.77–1.97)	2.23 (0.95–5.26)
Equol			
Tertile 1 (<3.75)	1.00 (reference)	1.00 (reference)	1.00 (reference)
Tertile 2 (3.75 to <9.73)	0.90 (0.44–1.85)	0.95 (0.71–1.27)	1.12 (0.64–1.97)
Tertile 3 (≥9.73)	0.54 (0.25–1.16)	0.76 (0.53–1.09)	0.74 (0.39–1.40)
Genistein			
Tertile 1 (<12.35)	1.00 (reference)	1.00 (reference)	1.00 (reference)
Tertile 2 (12.35 to <50.00)	0.58 (0.26–1.31)	1.50 (0.93–2.41)	0.70 (0.34–1.45)
Tertile 3 (≥50.00)	0.70 (0.17–2.85)	1.37 (0.76–2.46)	0.70 (0.29–1.69)
Males			
Enterolactone			
Tertile 1 (<160.53)	1.00 (reference)	1.00 (reference)	1.00 (reference)
Tertile 2 (160.53 to <621.68)	1.48 (0.70–3.13)	1.29 (0.89–1.88)	1.20 (0.65–2.23)
Tertile 3 (≥621.68)	0.50 (0.23–1.13)	0.94 (0.62–1.42)	0.89 (0.44–1.82)
Enterodiol			
Tertile 1 (<21.52)	1.00 (reference)	1.00 (reference)	1.00 (reference)
Tertile 2 (21.52 to <70.27)	1.31 (0.60–2.87)	1.40 (0.99–1.99)	1.05 (0.56–1.98)
Tertile 3 (≥70.27)	0.95 (0.39–2.28)	1.06 (0.73–1.54)	0.93 (0.47–1.84)
Daidzein			
Tertile 1 (<24.22)	1.00 (reference)	1.00 (reference)	1.00 (reference)
Tertile 2 (24.22 to <108.62)	0.63 (0.23–1.76)	1.28 (0.87–1.88)	1.72 (0.82–3.59)
Tertile 3 (≥108.62)	1.14 (0.24–5.40)	1.87 (0.95–3.65)	1.79 (0.68–4.72)
O-Desmethylangolensin			
Tertile 1 (<1.04)	1.00 (reference)	1.00 (reference)	1.00 (reference)
Tertile 2 (1.04 to <9.06)	1.59 (0.65–3.87)	0.90 (0.69–1.16)	0.94 (0.50–1.76)
Tertile 3 (≥9.06)	0.75 (0.21–2.68)	0.70 (0.46–1.04)	0.83 (0.38–1.82)
Equol			
Tertile 1 (<3.75)	1.00 (reference)	1.00 (reference)	1.00 (reference)
Tertile 2 (3.75 to <9.73)	1.07 (0.64–1.79)	1.06 (0.74–1.51)	0.95 (0.49–1.82)
Tertile 3 (≥9.73)	1.22 (0.66–2.26)	1.29 (0.92–1.81)	1.18 (0.62–2.27)
Genistein			
Tertile 1 (<12.35)	1.00 (reference)	1.00 (reference)	1.00 (reference)
Tertile 2 (12.35 to <50.00)	1.55 (0.61–3.93)	0.90 (0.63–1.29)	0.51 (0.30–0.87) *
Tertile 3 (≥50.00)	1.78 (0.68–4.62)	0.81 (0.51–1.27)	0.51 (0.25–1.02)

^a^ Adjusted for age, race, education, marital status, occupation, family income, body mass index, physical activity, alcohol use, smoking status, depressive symptoms, diabetes, hypertension, use of female hormones (only in females), C-reactive protein (mg/dL), caffeine intake (mg/day), and the other five phytoestrogens (tertiles). * *p* < 0.05.

## Supplementary Materials

The following are available online at https://www.mdpi.com/2072-6643/12/7/2103/s1, Table S1. The classifications of the covariates.
