# Supplementary material for: Associations of Urinary Phytoestrogen Concentrations with Sleep Disorders and Sleep Duration among Adults
Source: Nutrients. 2020 Jul 16;12(7):2103. doi: 10.3390/nu12072103 (PMC7400948; doi:10.3390/nu12072103)
Supplement: Supplementary file 1 [file nutrients-12-02103-s001.pdf]

Table 1. The classifications of covariates.

Covariates	Classifications
**Gender**	Female; Male
**Age (year)**	18–39; 40–59; ≥60
**Marital status**	Never married/widowed/divorced/separated; Married/living with partner
**Race**	Mexican American; Non-Hispanic White; Non-Hispanic Black; Other Hispanic; Other race
**Occupation**	No work; Regular night or evening shift/rotating shift/other; Regular daytime schedule
**Family income/year**	$20,000 and over; Below $20,000
**Educational level**	High school; Above high school; Below high school;
**Body mass index**	<18.5 kg/m^2; 18.5 to <25 kg/m^2; 25 to <30 kg/m^2; ≥30 kg/m^2
**Smoked at least 100 cigarettes in life**	No; Yes
**Had at least 12 alcohol drinks/year**	No; Yes
**Use of female hormones**	No; Yes
**Physical activity**	Moderate; Vigorous; Other
**C-reactive protein (mg/dL)**	Continuous
**Caffeine intake (mg/d)** [a]	Continuous
**Hypertension** [b]	No; Yes
**Depressive symptoms** [c]	No; Yes
**Diabetes** [d]	No; Yes

[a] Caffeine intake was the mean dietary caffeine intake through two 24-h dietary data.

[b] Participants whose mean diastolic (or systolic) blood pressure ≥80 (or ≥130) mmHg, or self-reported doctor diagnosis, or currently taking prescribed for high blood pressure were considered as having hypertension.

[c] Participants with the score of Patient Health Questionnaire-9 ≥10 were classified as having depressive symptoms.

[d] Diabetes was identified as self-reported doctor diagnosis.
